# Emerging PPAR**γ**-Independent Role of PPAR**γ** Ligands in Lung Diseases

**DOI:** 10.1155/2012/705352

**Published:** 2012-06-18

**Authors:** Ajit A. Kulkarni, Collynn F. Woeller, Thomas H. Thatcher, Sesquile Ramon, Richard P. Phipps, Patricia J. Sime

**Affiliations:** ^1^Division of Pulmonary and Critical Care, Department of Medicine, University of Rochester School of Medicine and Dentistry, Box 692, Rochester, NY 14642, USA; ^2^The Lung Biology and Disease Program, University of Rochester School of Medicine and Dentistry, Box 692, Rochester, NY 14642, USA; ^3^Department of Environmental Medicine, University of Rochester School of Medicine and Dentistry, Box 692, Rochester, NY 14642, USA; ^4^Department of Microbiology and Immunology, University of Rochester School of Medicine and Dentistry, Box 692, Rochester, NY 14642, USA; ^5^Department of Oncology, University of Rochester Medical Centre, USA

## Abstract

Peroxisome proliferator activated receptor (PPAR)-**γ** is a nuclear hormone receptor that is activated by multiple agonists including thiazolidinediones, prostaglandins, and synthetic oleanolic acids. Many PPAR**γ** ligands are under investigation as potential therapies for human diseases. These ligands modulate multiple cellular pathways via both PPAR**γ**-dependent and PPAR**γ**-independent mechanisms. Here, we review the role of PPAR**γ** and PPAR**γ** ligands in lung disease, with emphasis on PPAR**γ**-independent effects. PPAR**γ** ligands show great promise in moderating lung inflammation, as antiproliferative agents in combination to enhance standard chemotherapy in lung cancer and as treatments for pulmonary fibrosis, a progressive fatal disease with no effective therapy. Some of these effects occur when PPAR**γ** is pharmaceutically antagonized or genetically PPAR**γ** and are thus independent of classical PPAR**γ**-dependent transcriptional control. Many PPAR**γ** ligands demonstrate direct binding to transcription factors and other proteins, altering their function and contributing to PPAR**γ**-independent inhibition of disease phenotypes. These PPAR**γ**-independent mechanisms are of significant interest because they suggest new therapeutic uses for currently approved drugs and because they can be used as probes to identify novel proteins and pathways involved in the pathogenesis or treatment of disease, which can then be targeted for further investigation and drug development.

## 1. PPARs and PPAR Ligands

 Peroxisome proliferator activated receptors (PPARs) are a family of ligand-binding nuclear hormone receptors that are involved in multiple important regulatory pathways including fat metabolism, cell differentiation, proliferation, apoptosis, and inflammation. The first to be identified was PPAR*α*, the transcription factor responsible for the upregulation of peroxisome proliferation by fatty acids and certain hypolipidemic fibrate drugs. Three PPARs have now been identified, *α*, *β*/*δ*, and *γ*, encoded by three separate genes. PPAR*γ* is expressed as two different isoforms, PPAR*γ*1 and PPAR*γ*2, with different transcription start sites. Their detailed domain structure, characteristics, and mode of action have been discussed in detail elsewhere [1, 2].

 PPARs have two functional domains, a ligand-binding domain (LBD) and a DNA Binding domain (DBD). Both of these domains are highly conserved in all three receptors. The ligand-binding pocket of the LBD is relatively large and is assumed to bind to a wide range of ligands [2, 3]. Endogenous ligands of the PPARs include fatty acids and fatty acid metabolites; as a result, PPARs are believed to act in part as lipid sensors. PPARs also bind leukotrienes and prostaglandins such as 15d-PGJ_2_, which can have powerful regulatory effects on differentiation and immune responses [4]. At high enough concentration, the PPARs exhibit relatively nonselective binding for fatty acids. However, the significance of this is unclear because the concentration of any ligand in the cellular microenvironment has not been determined [2–5].

 Synthetic ligands for all three receptors have been identified and developed. The thiazolidinediones (TZDs) (rosiglitazone, pioglitazone, troglitazone, and ciglitazone) were developed as hypoglycemic agents in the 1980s [6], and the first clinical trial of a TZD as an insulin sensitizer was reported in 1991 [7]. Only after the TZDs were in clinical use were they recognized as ligands of PPAR*γ*, activating gene transcription to upregulate expression of adipocyte genes and induce adipocyte differentiation [8]. Although TZDs bind mainly to PPAR*γ*, select TZDs activate other PPARs as well [2, 9, 10].

The fibrate drugs (clofibrate, fenofibrate, gemfibrozil, ciprofibrate) have been in clinical use since the 1930s as cholesterol-lowering agents, but it was not until 1995 that it was discovered that they acted via PPARs [11]. The fibrates act most strongly on PPAR*α* but have activity against PPAR*β*/*δ* and *γ* as well. For example, benzafibrate is a pan-agonist with similar activity against all three PPAR isoforms. Other dual or pan-PPAR ligands have been developed including KRP-297, a dual PPAR*α*/*γ* agonist. Some nonsteroidal anti-inflammatory drugs (NSAIDs) (ibuprofen, indomethacin, and fenoprofen) weakly activate both PPAR*γ* and *α* [12].

The triterpenoids are a newly recognized class of natural exogenous PPAR*γ* ligands initially identified in herbal medicines. These compounds are being studied in their native forms and as synthetic derivatives with increased stability, potency, and selectivity. Ursolic acid, a plant triterpenoid, activates PPAR*α* and has been shown to regulate hepatic lipid metabolism [13]. Oleanolic acid and its naturally occurring derivative 2-cyano-3,11-dioxo-18-olean-1,12-dien-30-oate (CDDO) are potent PPAR*γ* agonists [14, 15].

## 2. PPAR**γ** and Lung Disease

Beyond its classical role in regulating fat metabolism, PPAR*γ* plays a role in regulating cell differentiation and inflammation. It is thus of high interest as potential target for therapies for diseases involving dysregulated inflammation or differentiation. Chronic inflammation due to cigarette smoking and exposure to other environmental toxicants is a contributing factor to numerous lung diseases including COPD (emphysema and chronic bronchitis), asthma, lung cancer, and fibrosis. PPAR*γ* expression is dysregulated in patients with cystic fibrosis, sarcoidosis [16], COPD [17, 18], and acute lung injury [19]. Often, and particularly with respect to COPD and fibrosis, current therapies only treat the symptoms and do not modify the course of the underlying disease [17, 20]. Thus, there is great interest in understanding the molecular pathways of chronic lung disease and of using PPAR*γ* ligands both as tools to probe these pathways and as potential therapies.

The lung represents a particularly useful target for this research as (a) many lung diseases involve acute or chronic inflammation or cell proliferation, which are processes targeted by PPAR*γ* ligands, (b) drugs can be delivered by inhalation, possibly sparing systemic side effects like heart disease and weight gain, and (c) PPAR*γ* ligands appear particularly effective against lung fibrosis, a disease for which effective therapy can be challenging. There are numerous studies demonstrating the potential role of PPAR*γ* and PPAR*γ* agonists in regulating or treating lung diseases including cancer, fibrosis, and diseases of chronic inflammation. Surprisingly, many of the most interesting effects appear not to require PPAR*γ* itself as a transcription factor.

## 3. PPAR**γ**-Independent Effects of PPAR**γ** Ligands

**Figure 1:**
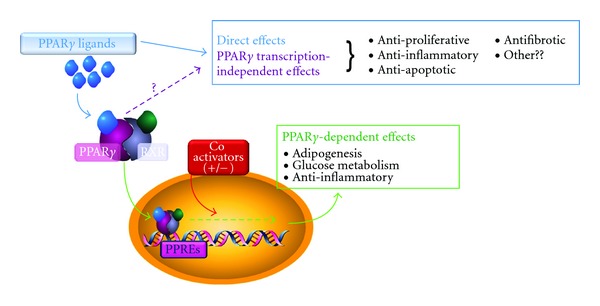
PPAR*γ* ligands have multiple PPAR*γ*-independent effects. In the classical PPAR*γ*-dependent pathway, ligand-bound PPAR*γ* forms a heterodimer with RXR and binds to PPAR*γ*-response elements (PPREs) which leads to modulation of transcription. However, PPAR*γ* ligands also exhibit direct effects that do not involve transcriptional activation by PPAR*γ*/RXR. These direct effects may involve PPAR*γ* protein interacting with PPAR*γ* ligands in a “non-classical manner” (not involving RXR or PPRE) or may be completely independent of PPAR*γ* (functioning even in the complete absence of PPAR*γ* protein, i.e., direct effects). PPAR*γ*-independent effects can alter multiple cellular programs including regulation of differentiation, inflammation, apoptosis and may be of significant therapeutic interest.

 Because they play important roles in multiple critical cellular pathways including differentiation, fat metabolism, proliferation, and control of inflammation, PPAR*γ* and its ligands have come under increasing scrutiny. However, some effects of PPAR*γ* ligands appear to be PPAR*γ*-independent—that is, they do not require PPAR*γ*-dependent transcriptional activation and can occur when PPAR*γ* is absent or functionally inactivated (Figure 1). The independent effects of PPAR*γ* were first recognized in 2000 by Thieringer et al. [21] who reported that 15d-PGJ_2_ had anti-inflammatory activity on human peripheral blood monocytes *in vitro* that was mediated by a PPAR*γ*-independent mechanism. More recently, it was reported that 15d-PGJ_2_ and ciglitazone induce apoptosis in both normal and malignant human B lymphocytes independent of PPAR*γ* activation [22]. It should be noted that PPAR*γ*-independent effects can be determined *in vitro* with relative confidence by using PPAR*γ*-antagonists, gene deletions, overexpression of dominant-negative mutants, or siRNA. However, it is more difficult to discriminate between PPAR*γ*-dependent and -independent effects *in vivo* because complete PPAR*γ* deletion is embryonically lethal [14].

Emerging reports demonstrating PPAR*γ*-independent effects of PPAR*γ* ligands are igniting interest in understanding the mechanisms responsible for the PPAR*γ*-independent effects of these compounds. Although they were initially identified as ligands of PPAR*γ*, their off-target or direct effects are important as these ligands regulate numerous signaling components that are independent of the classical PPAR*γ* pathway. These effects are important for two reasons. First, as PPAR*γ* ligand-based therapies enter into clinical testing, it will be critical to understand both their PPAR*γ*-dependent and -independent effects, so that their mechanism of action and potential side effects can be evaluated [23]. Second, PPAR*γ* ligands with PPAR*γ*-independent effects can be used to identify novel metabolic and regulatory pathways that play a role in human disease and may lead directly to novel therapeutic uses of existing drugs, or to development of new drugs that target these novel pathways uncovered by investigating PPAR-independent effects of these ligands.

## 4. Mechanism of PPAR**γ**-Independent Action of PPAR**γ** Ligands

A key question that must be answered as new PPAR*γ*-ligand-based therapies are developed is what the mechanisms and targets of the PPAR*γ*-independent effects of these compounds are. Current evidence suggests that the triterpenoids CDDO (and its derivatives) and the prostaglandin 15d-PGJ_2_ contain electrophilic carbon atoms that mediate their effects. These carbons can form covalent bonds with free sulfhydryl groups in cellular proteins via the Michael addition reaction, which can then alter the function of the protein. For example, 15d-PGJ_2_ can bind p50, which changes its DNA-binding activity [24]. CDDO is a strong electrophile that binds promiscuously to cellular proteins thus making its mechanism of action complex. A recent study in human embryonic kidney cells identified 577 cellular proteins that could bind to CDDO. The analysis was unable to discriminate between high abundance-low affinity targets and low abundance-high affinity targets, and many of these proteins may interact with CDDO without changing their function [25]. Nevertheless, direct protein binding by CDDO and other electrophilic PPAR*γ* ligands likely plays a key role in their function, and efforts are underway to identify specific binding partners in specific cell types and disease models.

One target of CDDO and 15d-PGJ_2_ is cellular antioxidant defenses. Both CDDO and 15d-PGJ_2_ bind glutathione, a key cellular antioxidant. By binding glutathione, CDDO and 15d-PGJ_2_ appear to deplete cellular stores of free glutathione, leading to increased oxidative stress and upregulation of antioxidant genes [26–28]. In some but not all cell types, CDDO also binds Keap-1, which then undocks from its binding partner, the transcription factor Nrf2. Nrf2 is then free to translocate to the nucleus where it is a master regulator of the oxidative stress response. Oral dosing with a CDDO Imidazole derivative prevented emphysema-like changes in lung structure in mice exposed to chronic cigarette smoke, and this effect was abrogated in Nrf2-knockout mice [29]. Activation of antioxidant defense systems may be one mechanism by which PPAR*γ* ligands can be used to prevent or treat diseases, especially cancer.

Glucocorticoid receptors (GRs) are also being explored as possible targets of PPAR*γ*-independent signaling mechanism. Initial work done by Ialenti et al. showed that rosiglitazone, and ciglitazone decrease IL-6 production in E8.2/GR3 and J774 cells, which is blocked by the GR inhibitor RU486 [30]. Rosiglitazone, pioglitazone and ciglitazone increased GR translocation to the nucleus in HeLa, A549, and PPAR*γ*-deficient fibroblasts [31]. In addition, evidence was found for rosiglitazone-induced GR recruitment of the transcriptional coactivators SRC-1 and NCoR in HeLa cells [31]. It was also reported in human bronchial cells that the antiproliferative effects of rosiglitazone and troglitazone were mediated by calcium channel signaling through the GPR40 receptor [32].

It should also be noted that it is not always possible to determine whether an effect that is independent of PPAR*γ*-regulated transcriptional control is also independent of the presence of PPAR*γ* protein (Figure 1). PPAR*γ* ligands may have “direct” effects that bypass classical RXR/PPRE interactions but which nevertheless require a functional PPAR*γ* protein acting in a nonclassical fashion (possibly as a result of covalent modification). Some studies make use of a small molecule inhibitor of PPAR*γ*, GW9662, that irreversibly binds to and inactivates the ligand-binding pocket of PPAR*γ*, while other studies have been performed in cells that are genetically deficient in PPAR*γ*. In these cases, it seems reasonable to conclude that the effects of the PPAR*γ* ligands are truly PPAR*γ*-independent. However, other studies utilize a dominant-negative PPAR*γ* construct that can bind ligands and the PPRE but cannot recruit transcriptional co-factors, or ligand derivatives that do not activate PPRE reporter constructs. In these cases, it can not be ruled out that PPAR*γ* ligands bind PPAR*γ* protein and carry out their function by a nonclassical pathway that bypasses RXR and the PPRE. Ligands may also act via different pathways in different cell types. More study is needed to understand these PPAR*γ*-independent mechanisms.

## 5. Inflammation

In the lung, PPAR*γ* is expressed in endothelial cells, fibroblasts, smooth muscle cells, airway epithelial cells, and resident alveolar macrophages, as well as infiltrating leukocytes including neutrophils, eosinophils, and mast cells [33, 34]. PPAR*γ* expression has been identified in multiple lung cell lines including A549, BEAS-2B, and NCI-H292 [35].

Clinical evidence suggests that PPAR*γ* and PPAR*γ* ligands are involved in regulating inflammatory responses in the lung [36, 37], and chronic or dysregulated inflammation is an important pathologic feature in many lung diseases including asthma, COPD, sarcoidosis, and pulmonary fibrosis. Expression of PPAR*γ* is increased in lung tissue from asthmatic patients, specifically localized in mucosal, epithelial, and smooth muscle cells and is associated with the severity of inflammation [34]. PPAR*γ* expression was decreased in patients with severe pulmonary hypertension, particularly around plexiform lesions [38].

Troglitazone, ciglitazone, and 15d-PGJ_2_ strongly inhibited LPS-induced secretion of TNF*α* and IL-6 and downregulated expression of Cox-2 and iNos in mouse macrophages. PPAR*γ*-deficient macrophages responded similarly to wild-type control, suggesting a PPAR*γ*-independent signaling mechanism [39]. Troglitazone and 15d-PGJ_2_ also activated PI3K signaling independently of PPAR*γ* in A549 cells (a human lung epithelial carcinoma line) via increased ERK phosphorylation and ERK/MAPK signaling, leading to increased production of Cox-2 and PGE_2_ [40]. Troglitazone and rosiglitazone promoted proliferation in nonmalignant human bronchial epithelial cells by a PPAR*γ*-independent mechanism, while 15d-PGJ_2_ blocked proliferation by a PPAR*γ*-dependent mechanism [32]. Recently, we reported that 15d-PGJ_2_ and CDDO inhibited the silica-induced inflammatory response of primary human lung fibroblasts via a largely PPAR-independent pathway attributed to the electrophilic properties of non-TZD ligands of PPAR*γ* [41].

In light of their anti-inflammatory properties *in vitro*, animal models have been used to explore the potential of PPAR*γ* ligands as therapeutic agents in inflammatory lung diseases. Oral administration of ciglitazone decreased cell infiltration, epithelial dysplasia, and IL-2, IL-4, and IFN*γ* production in a mouse model of allergic airway inflammation [42]. A similar effect was observed with intranasal administration of ciglitazone, with decreased cell infiltration in the lung, particularly that of eosinophils, and reduced airway remodeling [43]. Other TZDs such as rosiglitazone also decreased neutrophil and eosinophil infiltration and reduced airway hyperresponsiveness to methacholine [44]. Ciglitazone also decreased local inflammation in the lung in a model of *S. pneumoniae-*induced pneumonia, with concurrent reductions in TNF*α*, IL-6, IL-12p70, and IFN*γ* secretion [45]. However, it is difficult to attribute the effects of the ligands in *in vivo* studies to PPAR*γ*-dependent or -independent mechanisms, and further study is needed.

## 6. Lung Cancer and PPAR**γ** Ligands

Lung cancer accounts for more cancer-related deaths than any other type of cancer worldwide [46]. In 2010, there were 157,300 lung cancer-related fatalities in the United States alone [47]. As a disease, lung cancer can be divided into two main categories, nonsmall cell lung cancer (NSCLC), which comprises around 85% of all lung malignancies, and small cell lung cancer (SCLC), which comprises the other 15% [48]. NSCLC can be further classified into three subgroups, large cell cancer, adenocarcinoma, and squamous cell carcinoma, depending upon cell type and histological classification. As lung cancer is a substantial cause of human suffering and death, a multitude of therapeutic approaches have been employed. Many therapeutic options are actively being pursued including several recent investigations that utilize PPAR*γ* ligands as treatments [49, 50]. While recent work demonstrates PPAR*γ* ligands have beneficial PPAR*γ*-dependent and -independent properties in treating malignancies, emerging insights into PPAR*γ*-independent functions in lung cancer are highlighted here.

### 6.1. *In Vitro* Models of Lung Cancer and PPAR**γ**  Ligands

PPAR*γ* ligands including the TZDs, 15d-PGJ2, and triterpenoids (CDDO and its derivatives) have been investigated in lung cancer cells *in vitro*. Troglitazone dramatically reduces cell proliferation of A549 cells, a human lung adenocarcinoma cell line, at concentrations of 10 to 40 *μ*M. Because PPAR*γ* activity, determined by reporter assays, reached a maximal level at 15 *μ*M while the beneficial reductions in cell proliferation continued up to 40 *μ*M, the authors suggested that troglitazone had desirable PPAR*γ*-independent effects at elevated doses [51]. Troglitazone decreased expression of the cell cycle regulatory protein cyclin D1 and increased the number of cells that were growth-arrested in the G_1_/G_0_ phase of the cell cycle [51]. Recently, a troglitazone analog, delta2-Troglitazone, that does not stimulate PPAR*γ*-dependent transcription, was shown to increase proteasomal degradation of cyclin D1 in breast cancer cells [52]. This suggests that troglitazone, at least in part, affects cyclin D1 expression independent of PPAR*γ*. Cyclin D1 is highly expressed in many types of tumors, including the lung [53, 54]. Troglitazone also modifies ERK-1/2 signaling in NSCLC cells [51, 55–57] and triggers apoptosis in two lung cancer cell lines, but not in a control, nonmalignant line, underscoring the potential of TZDs to specifically target tumor cells [56]. In A549 cells, troglitazone directly interacts with the epidermal growth factor receptor (EGFR), binding the EGFR and augmenting its internalization into the lysosome where it is subsequently degraded [58]. The reduction in EGFR protein levels leads to a loss of EGFR signaling activity, which consequently halts cell proliferation. Interestingly, EGFR mutations are some of the most common mutations found in lung cancer patients [48].

Pioglitazone and rosiglitazone also show a number of beneficial effects in attenuating growth of lung cancer cells *in vitro*. In 2006, Han and Roman determined that rosiglitazone had significant PPAR*γ*-dependent and -independent effects on blunting NSCLC cell growth [59]. In this study, rosiglitazone impaired proliferation of NSCLC cells by increasing expression of the tumor suppressor phosphatase PTEN (phosphatase and tensin homolog), reducing Akt and p70S6K phosphorylation, and increasing phosphorylation of AMPK. The irreversible PPAR*γ* inhibitor GW9662 reversed the effects on PTEN expression and Akt phosphorylation but not AMPK and p70S6K phosphorylation, suggesting a mixture of PPAR*γ*-dependent and -independent effects. AMPK is a master regulator of energy metabolism and regulates mammalian target of rapamycin (mTOR) mediated cell growth, proliferation, and protein synthesis pathways. Phosphorylation of AMPK inhibits mTOR signaling to reduce proliferation and protein synthesis through a reduction of p70S6K phosphorylation. Interestingly, rosiglitazone enhanced the effects of rapamycin on halting NSCLC cell growth, accentuating the potential for combination therapies. The mechanism whereby rosiglitazone activates AMPK phosphorylation is still unclear.

Pioglitazone and rosiglitazone also decreased production of the prostaglandin PGE_2_ in both A549 and A427 NSCLC cell lines. Increased levels of PGE_2_ stimulate proliferation and prosurvival pathways in numerous malignancies, including NSCLC, suggesting that decreasing PGE_2_ production may reduce cancer cell growth. The effects of the agonists were not reversed by either GW9662 or by expression of a dominant-negative PPAR*γ*, demonstrating that the effects were PPAR*γ*-independent. Investigation into the mechanism determined a corresponding increase in the expression of 15-hydroxy prostaglandin dehydrogenase (15-PGDH), which converts PGE_2_ into a biologically inactive 15-keto prostaglandin derivative [60]. Rosiglitazone also inhibited expression of the *α*4 nicotinic acetylcholine receptor (*α*4-nAcR) in a PPAR*γ*-independent manner in three different NSCLC cell lines [61]. Nicotine stimulates proliferation of NSCLC cells through activation of *α*4-nAcR. Rosiglitazone activated Erk-1/2 signaling to prompt the tumor suppressor protein p53 to inhibit *α*4-nAcR expression. The effects of rosiglitazone treatment on *α*4-nAcR expression were independent of PPAR*γ* as demonstrated by depletion of PPAR*γ* protein by PPAR*γ* siRNA as well as through the use of GW9662 [62]. Rosiglitazone and GW1929, another TZD, also inhibit fibronectin expression by tumor cells *in vitro* [63]. Fibronectin expression and extracellular deposition promote tumor cell interactions.

An interesting non-lung study demonstrated that TZDs reduce expression of the oncogenic transcription factor FoxM1 in hepatoma cells [64]. Regulation of FoxM1 expression by TZDs was independent of PPAR*γ* expression, as expression of FoxM1 was reduced by TZDs even in the presence of PPAR*γ* siRNA. Because FoxM1 is often elevated in NSCLC, further investigation to see if TZDs can also reduce FoxM1 expression in lung cancer may be worthwhile.

The endogenous PPAR*γ* ligand, 15d-PGJ_2_, has also been shown to have beneficial effects in treating lung cancer cells. 15d-PGJ_2_ promotes apoptosis of A549 cells by activating caspase 3 and attenuating expression of cyclin D1 in a PPAR*γ*-independent manner [65]. 15d-PGJ_2_ also increased production of reactive oxygen species (ROS) in lung cancer cells while reducing intracellular glutathione levels [66]. The effects could not be reversed by a PPAR*γ* siRNA or the antagonist GW9662 but was reversible with quercetin, a powerful antioxidant, suggesting that 15d-PGJ_2_ modulated the redox state of the cell. Interestingly, 15d-PGJ_2_ has been shown to covalently attach to glutathione and lead to its oxidation [67], which may account for the loss of glutathione seen in the studies. 

The novel triterpenoid CDDO and its synthetic derivatives CDDO-Methyl ester (CDDO-Me) and CDDO-Imidazole (CDDO-Im) have also been investigated as chemotherapeutic agents in treating lung cancer. CDDO-Im promoted apoptosis in A549 and H358 human lung cancer cell lines in a dose-dependent manner with a rapid decrease in STAT5 phosphorylation [68]. STAT5, a phosphorylation-dependent transcription factor in the JAK/STAT pathway, is involved in the transcription of proliferation and prosurvival genes. CDDO-Im induced expression of SOCS-1 (a STAT inhibitor) and SHP-1 (a phosphatase that targets STAT5) in as little as 30 minutes. Because SOCS-1 and SHP-1 are both regulated by the antioxidant response transcription factor, Nrf2, the authors speculate that CDDO-Im activates Nrf2 by binding to its regulatory partner Keap-1 [68].

### 6.2. Animal Models of Lung Cancer and PPAR**γ** Ligands

Although it has been difficult to dissect out the specific PPAR*γ*-independent functions of the PPAR*γ* ligands from their PPAR*γ*-dependent functions *in vivo*, the ability of PPAR*γ* ligands to inhibit tumor cell growth *in vitro* has led to a number of significant studies in animal models. Ciglitazone, which inhibits proliferation of A549 cells *in vitro* by a PPAR*γ*-independent mechanism as discussed above, also significantly reduced A549 tumor weights in the nude mouse xenograft model, with concomitant reduction in cyclin D1 and increased expression of the cell cycle inhibitor p21 [51]. The xenograft model was also used to demonstrate that daily treatment with 15d-PGJ_2_ had potent antitumor effects against subcutaneous tumors comprised of A549 or H460 cells. Of note, 15d-PGJ_2_ significantly enhanced the effects of the chemotherapeutic drug docetaxel [65].

In the lung, Girnun et al. utilized a transgenic mouse model containing inducible activating mutations in either KRAS or epidermal growth factor (EGFR), two genes commonly mutated in human lung cancer [69]. Rosiglitazone, in combination with the standard chemotherapeutic agent carboplatin, significantly reduced tumor size by increasing apoptosis and decreasing cell proliferation. Importantly, neither drug alone had significant effects, and rosiglitazone did not enhance the myelosuppressive effects of carboplatin, suggesting that combination therapy would be safe and effective. Addition of rosiglitazone to a chemotherapeutic cocktail of hydrazine, selenium, and phenylbutyrate significantly reduced lung hyperplasia and adenoma in a mouse carcinogen model compared to the cocktail alone [70]. In another carcinogen-induced lung cancer model in mice, pioglitazone treatment had a clear preventative effect [71]. Mice given oral doses of pioglitazone 8 weeks after initial injection of carcinogen had no change in number of adenocarcinomas but a 64% decrease in tumor volume compared to control, along with a 35% reduction in the number of squamous cell carcinoma.

The CDDO derivatives CDDO-Me and CDDO-ethyl amide also inhibit lung cancer in preclinical mouse studies. In one study, mice were fed the CDDO derivatives starting one week after treatment with vinyl carbamate, a carcinogen that induces adenocarcinoma of the lung. The CDDO-treated mice had a dramatically reduced tumor burden [72]. The authors suggest that CDDO prevented tumor formation through upregulation of the antioxidant heme-oxygenase-1 (HO-1) and through inhibition of STAT phosphorylation leading to increased apoptosis, as demonstrated *in vitro*. In another recent study using the transplantable tumor model, Lewis lung carcinoma (LLC), CDDO-Me drastically reduced tumor size and inhibited myeloid-derived suppressor cells that are involved in the immune suppressive response against tumors [73]. These data are very intriguing as they suggest CDDO-Me may augment cancer vaccine therapies.

## 7. PPAR-Independent Effects of PPAR Ligands in Pulmonary Fibrosis

Fibrotic remodeling following injury and repair occurs in many tissues including the kidney, heart, skin, and liver [74]. Pulmonary fibrosis is characterized by pathologic remodeling of the lung, with thickening of interstitial spaces, deposition of collagen and other matrix proteins, contraction and stiffening of lung tissue, loss of alveolar architecture, reduced gas exchange and ultimately respiratory failure. Pulmonary fibrosis can be caused by a variety of insults including chronic inflammation and inhalation of particulates like asbestos and silica, radiation, drugs, and as a squeal of connective tissue diseases. Idiopathic pulmonary fibrosis (IPF) is a severe form of pulmonary fibrosis with no current therapy. The only “cure” is a lung transplant. The disease is progressive and the median time from diagnosis to death at 2.9 years is shorter than that for lung cancer [15].

 One of the key pathogenic processes in lung fibrosis is the differentiation and activation of fibroblasts and myofibroblasts to produce the components of scar tissue (hypercellularity, collagen, and other extracellular matrix proteins). The key pro fibrotic cytokine appears to be TGF*β*. Because of the central role of TGF*β* and myofibroblasts, there is intense interest in developing novel therapies that target these key players. Pirfenidone, a small orally active molecule that inhibits synthesis of TGF*β* and TNF*α* and attenuates growth of fibroblasts *in vitro*, was recently approved for clinical use in IPF in Europe, but not yet in the United States. The clinical benefits of pirfenidone appear to be small but significant in some studies [75]; no other therapies have been approved that specifically target the underlying cellular pathology of lung fibrosis.

### 7.1. *In Vitro* Models of Pulmonary Fibrosis and PPAR Ligands

There is a growing body of evidence that both natural and synthetic PPAR*γ* agonists have powerful antifibrotic effects *in vitro* [76], and these results are beginning to be translated to preclinical animal models. Typical experiments examine differentiation of human lung fibroblasts to myofibroblasts *in vitro* and associated changes in expression of profibrotic cytokines and matrix proteins. A variety of nontransformed human lung fibroblast (HLF) cell lines are used including fetal, neonatal, adult nonfibrotic, and adult fibrotic (derived from patient biopsies).

**Figure 2:**
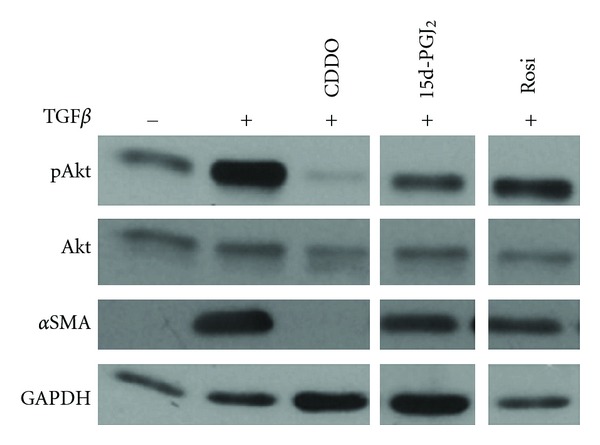
PPAR*γ* ligands inhibit TGF*β*-induced Akt phosphorylation and myofibroblast differentiation with varied potency. Primary human lung fibroblasts were treated with TGF*β* (5 ng/mL), alone or in combination with three PPAR*γ* ligands (CDDO (1 *μ*M), 15d-PGJ_2_ (5 *μ*M) and rosiglitazone (20 *μ*M)). Protein lysates were electrophoretically separated on the same gel, and representative lanes from a single experiment are shown here. The potency of a PPAR*γ* ligand to inhibit Akt phosphorylation corresponds to its ability to inhibit myofibroblast differentiation. While CDDO inhibits both Akt phosphorylation and *α*SMA potently, rosiglitazone is a weak inhibitor of both.

We and others reported that rosiglitazone and 15d-PGJ_2_ inhibited TGF*β*-driven myofibroblast differentiation of primary HLFs [77–80]. Expression of a dominant PPAR*γ* was able to reverse the inhibitory effect of rosiglitazone more effectively than 15d-PGJ_2_, suggesting that rosiglitazone can act via both PPAR*γ*-dependent and -independent mechanisms while 15d-PGJ_2_ acts predominantly via an independent mechanism [77]. Since primary HLFs express abundant PPAR*γ* and RXR proteins and are capable of PPAR*γ*-dependent transcriptional regulation [77], this suggested that that the antifibrotic effects of the PPAR*γ* agonists were mediated through both PPAR*γ*-dependent and -independent mechanisms. We also investigated CDDO, a triterpenoid originally identified in herbal preparations with anti-inflammatory properties. We determined that CDDO has an EC_50_ for inhibition of myofibroblast differentiation that is 20-fold lower than 15d-PGJ_2_ and 400-fold lower than rosiglitazone, and it acts independently of PPAR*γ* as confirmed by pharmacological and genetic approaches [78]. Recently, we reported that 15d-PGJ_2_ and CDDO inhibit TGF*β*-induced phosphorylation of phosphotidyl-inositol 3-kinase-protein kinase B (PI3K-Akt) and focal adhesion kinase (FAK), but not TGF*β* -induced p38-MAPK phosphorylation, and that the mechanism was independent of PPAR*γ* [79]. We also noted that there is a strong correlation between the ability of a PPAR*γ* ligand to inhibit Akt phosphorylation with its ability to suppress myofibroblast differentiation (Figure 2). We find that rosiglitazone is a weak inhibitor of TGF*β*-induced Akt phosphorylation (Figure 2) and may be therefore a poor choice as an antifibrotic treatment [79].

It is essential to note that the three main mechanisms of activation of myofibroblast differentiation—TGF*β*, mechanical stress, or adhesion and integrin activation—act via a common pathway in which FAK is autophosphorylated, leading to increased levels of the active kinase (phospho-FAK). It is conceivable that once TGF*β* activates myofibroblast differentiation, the increased deposition of extracellular matrix proteins would cause additional mechanical stress at the cell surface leading to sustained and continual activation of FAK. Since FAK itself upregulates myofibroblast differentiation, once TGF*β* initiates this process, sustained activation of FAK would be able to perpetuate the fibrotic response even in the absence of additional external profibrotic signals.

Several other laboratories have also investigated PPAR*γ* ligands in lung fibroblast differentiation and activation. Lin et al. reported that rosiglitazone inhibited migration, proliferation, and phenotypic differentiation of cultured fetal human lung fibroblasts induced by fetal bovine serum albumin. The inhibitory effect of rosiglitazone on myofibroblast differentiation, determined by expression of *α*-smooth muscle actin, was also PPAR*γ*-independent [81]. Troglitazone and ciglitazone were evaluated against normal human fetal lung fibroblasts and fibroblasts isolated from patients with idiopathic interstitial pneumonias. Both ligands inhibited proliferative responses of normal and fibrotic lung fibroblasts to platelet-derived growth factor (PDGF) and TGF*β* in a dose- and time-dependent fashion and arrested cells at the G_1_/G_0_ stage. Troglitazone and ciglitazone inhibited expression of cyclin D1, similar to their activity in the A549 adenocarcinoma cell line (see above). Interestingly, they also found that both troglitazone and ciglitazone efficiently inhibited collagen expression by myofibroblasts and that collagen synthesis was also inhibited by overexpression of a constitutively active form of PPAR*γ* [80]. Since cyclin D1 is a known downstream target of PI3K-Akt pathway, it is conceivable that these and other ligands of PPAR*γ* (15d-PGJ_2_ and CDDO) inhibit cyclin D1 and arrest fibroblast proliferation via a PPAR*γ*-independent mechanism involving Akt but inhibit collagen synthesis and other profibrotic functions by a PPAR*γ*-dependent mechanism.

Not all myofibroblasts in lung fibrosis are derived from preexisting lung resident fibroblasts. Some myofibroblasts may originate from lung epithelial cells via epithelial to mesenchymal transition (EMT). EMT is a process during which terminally differentiated epithelial cells transform into a mesenchymal phenotype and give rise to fibroblasts and subsequently to myofibroblasts. This process is believed to play an important role in normal repair following injury to alveolar epithelial cells (AECs) [74]. The *in vivo* significance of EMT is controversial, but it is well-described *in vitro* and is characterized by decreased expression of E-cadherin and increased expression of N-cadherin [82]. TGF*β* potently stimulates EMT and alters the morphology of human AECs. Tan et al. investigated whether PPAR*γ* ligands could inhibit TGF*β*-induced EMT in the A549 cell line, an adenocarcinoma of AEC origin. Rosiglitazone and ciglitazone rescued TGF*β*-mediated repression of E-cadherin via a PPAR*γ*-dependent mechanism but, at the same time, inhibited TGF*β*-induced increased N-cadherin expression independent of PPAR*γ* [82].

Myofibroblasts in lung fibrosis can also originate from circulating adult progenitor cells called fibrocytes. Fibrocytes are distinct from epithelial and endothelial cells, monocyte/macrophages, lymphocytes, dendritic cells, or tissue fibroblasts [83]. Work in the Strieter laboratory demonstrated that TGF*β* induces myofibroblast differentiation of fibrocytes harvested from human peripheral blood mononuclear cells as measured by *α*SMA expression [84]. Troglitazone inhibits TGF*β*-induced *α*SMA expression and myofibroblast differentiation, and this effect is not reversed by the PPAR*γ* inhibitor GW9662, indicating that the effects of troglitazone are PPAR*γ*-independent. TGF*β* signaling activates the Smad and MAP kinases pathways in fibrocytes, and PPAR*γ* agonists negatively regulate this process through a PPAR*γ*-independent pathway. Of special note, troglitazone inhibited JNK activation, which both inhibited myofibroblast differentiation and stimulated differentiation of fibrocytes to adipocytes (fat storage cells) [84]. Thus, pharmacological intervention with PPAR*γ* ligands may alter the fate of circulating myofibroblast precursors before they ever reach the lung.

### 7.2. PPAR**γ** Ligands in Animal Models of Lung Fibrosis

There are currently only a few published reports investigating PPAR*γ* ligands in animal models of fibrosis, although additional studies are in progress. Troglitazone given orally (200 or 400 mg/kg/day) inhibited bleomycin-induced lung fibrosis in mice, with reductions in lung collagen content and TGF*β* levels [80]. Rosiglitazone and 15d-PGJ_2_ were also tested in the mouse bleomycin model and reduced mortality, inflammation, cellular influx, and fibrosis [85]. To investigate the PPAR*γ* dependence of the effect, mice were cotreated with bisphenol A diglycidyl ether (BADGE), a PPAR*γ* antagonist. BADGE reversed the antifibrotic effects of rosiglitazone and 15d-PGJ_2_, suggesting that at least some of the antifibrotic activity of these compounds is mediated by a PPAR*γ*-dependent mechanism *in vivo* [86]. However, while BADGE has been reported to be a PPAR*γ* antagonist in some studies [85], it activates PPAR*γ* in others [87]. Interestingly, the stimulation of heme-oxygenase (HO-1) by 15d-PGJ_2_ in rat primary cortical neuron cultures was blocked by BADGE [88], but, in human lung fibroblasts, HO-1 induction was not blocked by another PPAR*γ* antagonist GW9662 [41]. This suggests that the same PPAR*γ* ligand may have dependent effects in some cell types and independent effects in others.

A different result was observed in a neonatal hyperoxia model. Premature infants requiring hyperoxia (100% O_2_) during the first weeks of life suffer from a high rate of bronchopulmonary dysplasia (BPD), characterized by mild inflammation and fibrosis, and failed alveolarization. In neonatal mice, hyperoxia leads to upregulation of TGF*β* and Wnt signaling and downregulation of PPAR*γ* signaling owing to reduction in both PPAR*γ* mRNA and protein [89, 90]. While rosiglitazone markedly prevented lung injury in hyperoxic neonates, the effect was reversed by administration of GW9662, suggesting a PPAR*γ*-dependent mechanism [89]. It should be noted that BPD is thought to represent a failure of the developmental program, and therefore the signals and mechanisms involved in neonatal hyperoxic fibrosis may be different than the signals involved in fibrosis of fully differentiated adult lungs, and it would be interesting to study PPAR*γ*-independent effects of electrophilic ligands of PPAR*γ* in the neonatal animal models [91].

Additional study is needed to characterize the *in vivo* mechanism of action of PPAR*γ* ligands. Because the bleomycin model is characterized by acute early inflammation, it is possible that the majority of the antifibrotic effects seen in these early studies are a result of PPAR*γ*-mediated inhibition of inflammation that precedes fibrosis. Therefore, it will be important to investigate the effects of PPAR*γ* ligands in fibrosis models that do not also have overt inflammation. CDDO and its derivatives have strong antifibrotic effects *in vitro* at much lower concentrations than TZDs but have not yet been investigated *in vivo* for lung fibrosis. However, it is clear that PPAR*γ* ligands have strong therapeutic potential in these studies regardless of whether the effects are dependent or independent of PPAR*γ*-mediated transcriptional regulation.

## 8. Other Pulmonary Diseases

Rosiglitazone attenuates pulmonary arterial hypertension in an ApoE null mouse model fed a high-fat diet [92]. This appears to be PPAR*γ*-dependent as mice with targeted deletion of the PPAR*γ* gene in smooth muscle cell tissue spontaneously developed pulmonary arterial hypertension [92]. Furthermore, under hypoxia-induced pulmonary hypertension conditions, rosiglitazone prevents and reverses pulmonary hypertension by downregulating Nox4 [93].

Acute promyelocytic leukemia (APL) is a subtype of acute myelocytic leukemia in which myeloid precursor cells grow aberrantly and can infiltrate into the pulmonary tract causing severe disease. APL is characterized by a genomic translocation fusing the PML tumor suppressor and the retinoic acid receptor *α* gene. While APL is normally treated with all-trans retinoic acid (ATRA), cells can become resistant to this therapy, thus novel drug cocktails are needed. CDDO has been shown to increase proapoptotic and differentiating effects of ATRA in APL-derived NB4 cells and partially reverse ATRA resistance in ATRA-resistant NB4-derived cells [94]. In a mouse model of APL initiated by a PML-RAR*α* transgene, CDDO and ATRA cotreatment significantly increased animal survival rates by compared to ATRA treatment alone. The PPAR*γ* selective antagonist T0070907, or PPAR*γ* siRNA only partially impaired the effects of CDDO treatment, suggesting both PPAR*γ*-dependent and -independent effects of CDDO in APL cells. Another report suggested beneficial, proapoptotic effects of ciglitazone in APL [95]. Ciglitazone treatment caused a significant increase in APL cell apoptosis demonstrated by DNA fragmentation analysis, increased active caspase-3, cleaved PARP, and decreased expression of the X-linked inhibitor of apoptosis protein. Notably, the effects of ciglitazone were reversed by treatment with GW9662, suggesting a PPAR*γ*-dependent mechanism.

## 9. Potential Therapies

Animal models clearly show extraordinary translational potential for PPAR*γ* ligands to serve as either preventative therapies or cotreatments for lung malignancies. Recent clinical studies also suggest favorable outcomes in lung cancer patients treated with PPAR*γ* ligands. In a clinical study encompassing patients with diabetes, lung cancer occurrence in patients receiving TZD therapy was decreased by 33% compared to patients not taking a TZD [96]. Notably, the decrease in lung carcinomas was still evident after confounding factors (i.e., age and race/ethnicity) were accounted for. At the present time, clinical trials are underway to investigate the effectiveness of TZDs in the prevention and/or treatment of lung cancer, either alone, or in combination with other chemotherapies. It is important to note that TZDs show both PPAR*γ*-dependent and -independent modes of action and further exploration of their mechanism of action is needed to elucidate their specific targets. CDDO and its derivatives have proapoptotic and antiproliferative effects on cancer cells *in vitro* and are currently being investigated in phase 2 clinical trials for treatment of cancer [97]. Further studies are required to determine if CDDO-Me will also be useful as a myelosuppressive agent in combination with chemotherapies.

Currently, there are no PPAR*γ* ligands in clinical trial for fibrotic lung diseases. However, there is evidence in other organ systems suggesting that PPAR*γ* ligands may have antifibrotic potential in human lung fibrosis. Intriguingly, kidney remodeling is a frequent complication of diabetes, and improvements in renal function have been noted in patients with type II diabetes treated with TZDs [98, 99]. The use of TZDs in clinical therapy may be limited by undesirable side effects. However, unlike diabetes, which can often be managed with alternate therapies, there are no current treatments for IPF and the median survival time after diagnosis is only 2-3 years, which may shift the risk-benefit assessment in favor of use.

In addition to lung cancer and fibrosis, pioglitazone and rosiglitazone are in ongoing clinical trials for asthma (e.g., NCT01134835 and NCT00614874), cystic fibrosis (e.g., NCT00322868 and NCT01060566), pulmonary hypertension (e.g., NCT00825266 and NCT00006071), and pulmonary arterial disease (e.g., NCT00153166 and NCT00064727). These studies will evaluate whether the *in vitro* anti-inflammatory effects of PPAR*γ* ligands can be translated to improved patient outcomes.

Current PPAR*γ* ligands act via both PPAR*γ*-dependent and -independent mechanisms, and the impact of both pathways must be considered in evaluating their efficacy in animal models and clinical trials. However, the existence of PPAR*γ*-independent effects creates the potential for development of novel non-PPAR-based therapies that access the independent pathways uncovered by PPAR*γ* ligands. In this event, currently available PPAR*γ* ligands with strong PPAR-independent effects, such as 15d-PGJ_2_ and CDDO, may play a more important role as probes for novel disease modifying pathways than as direct clinical treatments, paving the way for new molecules that target critical pathways (such as Akt, FAK, ERK, and JNK) without also activating PPAR*γ*.

It is interesting to note that some of the PPAR*γ*-independent effects of PPAR*γ* ligands are mediated by modulating the phosphorylation status of key regulatory enzymes such as Akt, FAK, ERK, and JNK. Imatinib mesylate (Gleevec), a broad-spectrum tyrosine kinase inhibitor currently approved to treat some cancers, has been tested in several small open-label trials in systemic sclerosis, a fibrosing disease that often involves the lung. In some studies, imatinib improved lung function and reduced skin contraction, although the number of adverse effects was high [100, 101]. However, in a randomized, placebo-controlled trial of patients with mild to moderate IPF, imatinib did not significantly reduce disease progression [29]. These studies demonstrate the clinical potential as well as limitation of antikinase treatments in lung fibrosis and encourage further exploration of the non-PPAR molecular targets of PPAR*γ* ligands.

## 10. Conclusion

Discovered serendipitously, the off-target, or PPAR*γ*-independent effects of PPAR*γ* ligands may prove as interesting and therapeutically useful as their PPAR*γ*-dependent effects. PPAR*γ* agonists have potent PPAR*γ*-independent effects *in vitro* and *in vivo*, regulating proinflammatory responses and acting to promote apoptosis and inhibit differentiation, which may be beneficial in treating cancer and fibrosing diseases. Clinical trials are underway, investigating the currently approved TZDs in novel diseases, and investigating novel agonists such as CDDO and its derivatives. Significant clinical progress can be expected in the next few years.

The most complex problem associated with the use of PPAR*γ* agonists as disease therapies is indeed the combination of potent PPAR*γ*-dependent and -independent activities they exhibit. This presents both multiple challenges and multiple opportunities for translational investigation. The PPAR*γ*-dependent and independent effects can be decoded with specificity using *in vitro* models, but the results are difficult to confirm *in vivo* because homozygous PPAR*γ* deletion is lethal. And, of course, patients will be subjected to both the dependent and independent effects of any new drugs, regardless of their primary mode of action *in vitro*. Thus, it will be critically important to carefully evaluate both the PPAR*γ*-dependent and -independent therapeutic effects and side effects of any new therapies.

Some PPAR*γ* ligands exhibit PPAR*γ*-dependent and -independent effects that act in the same direction, such as the antifibrotic activities of the TZDs. Unfortunately, the TZDs carry the risk of significant side effects including edema, weight gain, and cardiovascular effects. Troglitazone (Rezulin) has been withdrawn from the market due to hepatotoxicity [102], while rosiglitazone (Avandia) has been withdrawn in Europe and is under restriction in the US due to cardiovascular effects [103]. In the context of pulmonary disease, while there are alternative therapies for diabetes, there are no effective current therapies for lung fibrosis, and despite advances in chemotherapy, the five-year survival rate for NSCLC is only 15%. Thus, it is possible that these drugs will be revived for use in these deadly disease, either alone or in combination with other therapies. Alternatively, direct lung administration by inhalation may increase the effectiveness of the drugs while sparing the patient from systemic side effects, as is the case with inhaled steroids for asthma.

Also promising is the ability to use PPAR*γ* ligands as probes to uncover novel disease regulatory pathways which can then be targeted by new, specific therapies. Cyclin D1, Keap-1, Akt, and FAK are examples of disease targets that have been identified through the PPAR*γ*-independent effects of PPAR*γ* ligands. It is now possible, through mass spectrometry and other techniques, to determine exactly how these compounds bind to their targets and alter their function. This should allow the development of new compounds that have specific targeting activity against their new targets, while eliminating or reducing their affinity for PPAR*γ* and thus reducing or eliminating PPAR-dependent activity and its associated side effects. As these ligands enter clinical trials, there is an urgent need to understand their PPAR-dependent and -independent mechanisms of action for the future of targeted and personalized medicines.
